# Cross-sectional associations between fruit and vegetable intake and successful ageing across six countries: findings from the WHO Study on global AGEing and adult health (SAGE)

**DOI:** 10.1017/S1368980024001976

**Published:** 2024-10-21

**Authors:** Catherine M Milte, Karen E Lamb, Sarah A McNaughton

**Affiliations:** 1 Deakin University, Institute for Physical Activity and Nutrition (IPAN), School of Exercise and Nutrition Sciences, Burwood, VIC 3125, Australia; 2 Melbourne School of Population and Global Health, University of Melbourne, Parkville, VIC 3052, Australia; 3 Health and Well-Being Centre for Research Innovation, School of Human Movement and Nutrition Sciences, University of Queensland, St Lucia, QLD 4067, Australia

**Keywords:** Lifestyle and ageing, Dietary intake, Low- and middle-income countries

## Abstract

**Objective:**

This study develops successful ageing profiles across six low- and middle-income countries (LMIC) and examines associations with fruit and vegetable (F&V) intake.

**Design:**

A cross-sectional analysis was conducted in mid-aged and older adults from the WHO Study of Global Ageing. Participants without chronic disease, cognitive impairment, depression or disability and with good physical, cardiovascular and respiratory function were considered to have successfully aged. Associations between F&V intake (serves/d) and successful ageing were examined using log-binomial regression adjusting for key confounders.

**Setting:**

China, Ghana, India, Mexico, Russia and South Africa.

**Participants:**

A total of 28 785 men and women aged 50 years and over.

**Results:**

Successful ageing ranged from 4 % in Mexico to 15 % in China. After adjustment, only Ghana showed an association between fruit intake and successful ageing, with an inverse association identified (prevalence ratio (PR) = 0·87, 95 % CI 0·78, 0·98). An inverse association between vegetable intake and successful ageing was found in China (0·97, 0·95, 0·98) but no other country. An inverse association was shown for both China (0·98, 0·96, 0·99) and Ghana (0·92, 0·84, 1·00) when considering fruit and vegetables combined.

**Conclusions:**

Associations between F&V intake and successful ageing are inconsistent. Further studies on LMIC countries are needed to meet the challenges of the ageing population.

Increased life expectancy and declines in early mortality are leading to growth in the proportion of people aged ≥ 60 years worldwide, with the steepest increases forecast to occur in low- and middle-income countries (LMIC)^(1)^. Recognition of the importance that quality of life and good overall function accompany this increased life expectancy has led to increased interest in ‘successful ageing’^(2)^. Various concepts of successful ageing exist across social, psychological and medical sciences but can be defined as an older person living with the absence of disease and maintenance of physical and cognitive function, combined with the presence of life satisfaction, community participation and financial stability^(2)^. Healthy lifestyle behaviours are important factors in the maintenance of quality of life in older age^(3,4)^. Optimal nutrition in older age is important given the unique biological and social factors challenges that impact both nutritional intake and requirements in this life stage.

Intake of fruit and vegetables is considered a key component of a healthy diet informed by nutrition recommendations and dietary guidelines globally^(5,6)^. The WHO recommends consumption of 400 g of fruit and vegetables each day to prevent chronic disease in adults^(6)^. Although older people eat more servings of fruit and vegetables than younger adults, consumption is still low, with less than half of older adults meeting the recommended consumption levels^(7)^. Low rates of fruit and vegetable intake have been previously observed in LMIC in Eastern Europe and Central Asia^(8)^.

Previous studies have investigated associations between fruit and vegetable intake and risk of type 2 diabetes^(9)^, chronic disease including CVD^(10)^ and hip fracture^(11)^ with mixed findings. However, studies in this age group have often focused on individual disease outcomes, with less focus on investigation of holistic measures of health and function such as successful ageing. In addition, these studies have been primarily conducted in high-income countries. LMIC have experienced demographic and health transitions which combined with the coexistence of infectious disease and chronic disease burdens suggest that patterns of successful ageing and their determinants may differ from high-income countries^(12)^.

There is a clear need to study determinants of successful ageing in LMIC to inform evidenced-based strategies to support the rapidly growing ageing population observed in these unique populations. The aim of the current study was to develop successful ageing profiles and examine associations between fruit and vegetable intake and successful ageing across six LMIC (China, Ghana, India, Mexico, Russia and South Africa).

## Methods

### Data

This study used data from the first wave of the WHO Study of Global Ageing (SAGE). SAGE was designed to obtain greater understanding of ageing and health in LMIC and involves nationally representative samples from China, Ghana, India, Mexico, Russia and South Africa. Full details of the study and sampling have been described elsewhere^(13)^. In brief, multistage cluster random sampling was conducted in each country with all participants from households classified as ‘50+ year households’ invited to complete an individual face-to-face interview. Proxy respondents were identified for participants who could not complete the interview. Interviews were conducted between 2007 and 2010. Person-level analysis weights were calculated for each country; these included both a sample selection and a post-stratification factor, with the most recent population estimates provided by the national statistical offices in each country.

### Outcome: successful ageing

Successful ageing was measured based on seven components of physical and cognitive function^(14,15)^. Each component is briefly described below with additional details provided as Supplementary Information.

#### History of chronic disease

History of chronic disease was measured using self-reported prior diagnosis of the following diseases: stroke, angina and diabetes. In addition, four self-report items relating to common symptoms during the last 12 months were included as proxy measures of diagnoses of stroke or angina, respectively (see online Supplementary Table 1).

#### Physical function

Physical function was objectively measured using both a timed walk and grip strength to capture different aspects of physical capacity (namely locomotion and strength)^(16)^.

#### Timed walk

Participants were asked to walk 4 m over a flat and straight surface at their usual pace. Age- and sex-specific cut points were used, based on cut points from Oh-Park et al. 2010^(17)^ (online Supplementary Table 1).

#### Grip strength

A dynamometer was used to assess grip strength. The European Working Group on Sarcopenia classified cut points of < 30 kg for men and < 20 kg for women as poor grip strength^(18,19)^.

#### Respiratory function

Poor respiratory function was based on self-report of one of more of chronic lung disease, shortness of breath and coughing or wheezing (described in online Supplementary Table 1).

#### Cardiovascular function

The average of three measured systolic blood pressure (SBP) and diastolic blood pressure (DBP) results taken by the survey interviewer were used. The WHO cut points for high blood pressure (i.e. SBP ≥ 140 mmHg or DBP ≥ 90 mmHg) were used to classify people with poor cardiovascular function^(20)^.

#### Cognitive function

Cognitive function was assessed using an interviewer administered ten word verbal recall test, as well as self-report of difficulties with concentration or memory during the last 12 months.

#### Disability

Reporting either severe or extreme difficulty in one or more of the items from either the twelve-item WHO Disability Assessment Schedule (WHODAS) or fourteen questions relating to activities of daily living and instrumental activities of daily living was classified as presence of disability.

#### Mental health

Participants were reported to have good mental health if they did not report diagnosis with depression or symptoms for diagnosis of mild, moderate or severe depression over the last 12 months based on the International Classification of Disease 10th Revision, Diagnostic Criteria for Research (ICD-10-DCR)^(21)^.

#### Successful ageing

As defined elsewhere,^(14)^ participants were classified as successfully ageing if they had no history of chronic disease, good walking function, good grip strength, good respiratory function, good cardiovascular function, good cognitive function, no disability and good mental health. Participants were defined as not successfully ageing if they had poor outcomes for at least one of these components. In addition to the binary outcome, each of the eight components was summed in a successful ageing score, as described in online Supplementary Table 1. Lower scores indicate more successful ageing.

### Exposure: fruit and vegetable intake

As part of the questionnaire, participants were asked to separately report how many servings of fruit and vegetables they eat on a typical day. Country-specific examples of servings were provided. The current analysis considered the number of serves of fruit and vegetables per d reported as continuous variables. In addition, the average serves of fruit and vegetables per d were summed to create a combined measure of the total numbers of serves of fruit and vegetables per d. To avoid unrealistic values (e.g. eighty serves of vegetables per d for China), values greater than three times the sd from the mean were recoded to missing for each country. This resulted in maximum values of ten fruit serves and twenty vegetable serves per d for China, eight fruit serves and five vegetable serves per d for Ghana, four fruit serves and five vegetable serves per d for India, five fruit serves and five vegetable serves per d for Mexico and Russia, and five fruit serves and six vegetable serves per d for South Africa.

### Potential confounders

Age, gender, years of schooling, marital status, employment, urban/rural location and country-specific ethnicities were considered as potential confounders and adjusted for in the primary adjusted analyses (adjustment 1). In sensitivity analyses, total minutes of physical activity per week and total minutes of sedentary time per week, smoking status, alcohol consumption over the past 12 months and BMI were also included in the models (adjustment 2) (see online Supplementary Figures 1 and 2). See supplementary information for further details.

### Statistical analysis

Country-specific descriptive characteristics were calculated for each continuous (mean and sd) and categorical variable (*n* (%)) (Table 1). Descriptive characteristics for each element within the successful ageing score are presented in online Supplementary Table 2.

**Table 1.** Descriptive characteristics for eligible study participants by country

	China (*n* 12 999)					Ghana (*n* 4248)					India (*n* 6477)				
Variable	Mean	sd	Range	*n*	%	Mean	sd	Range	*n*	%	Mean	sd	Range	*n*	%
Successful ageing															
No				10 254	78·9 %				3867	91·0 %				6045	93·3 %
Yes				1917	14·8 %				343	8·1 %				386	6·0 %
Missing				828	6·4 %				38	0·9 %				46	0·7 %
Successful ageing score															
Missing	1·67	1·30	0–8	2295	17·7 %	2·27	1·42	0–7	399	9·4 %	2·69	1·58	0–8	496	7·7 %
Servings of fruits consumed on typical day															
Missing	2·4	2·2	0–10	1107	8·5 %	2·0	1·5	0–8	133	3·1 %	0·9	0·9	0–4	155	2·4 %
Servings of vegetables consumed on typical day															
Missing	6·99	3·95	0–20	678	5·2 %	2·01	0·83	0–5	148	3·5 %	1·93	0·83	0–5	82	1·3 %
Servings of fruits and vegetables consumed on a typical day	9·37	4·72	0–30	1179	9·1 %	4·00	1·97	0–13	230	5·4 %	2·76	1·37	0–9	211	3·3 %
Age (years)	63·11	9·39	50–96			64·32	10·67	50–114			61·74	8·95	50–105		
Gender															
Male				6101	46·9 %				2219	52·2 %				3272	50·5 %
Female				6898	53·1 %				2029	47·8 %				3205	49·5 %
Ethnicity^a^				*Han*: 12 566	96·7 %				*Akan*: 2047	48·2 %				*Scheduled tribe/caste:* 1469	22·7 %
				*Other*: 196	1·5 %				*Ewe*: 290	6·8 %				*No caste or tribe*: 1090	16·8 %
									*Ga-Adangbe:* 424	10·0 %				*Other:* 3885	60·0 %
									*Gruma:* 213	5·0 %					
									*Mole-Dagbon:* 107	2·5 %					
									*Other:* 1089	25·6 %					
Missing				237	1·8 %				78	1·8 %				33	0·5 %
Marital status															
No partner				2199	16·9 %				1823	42·9 %				1662	25·7 %
Partner				10 793	83·0 %				2403	56·6 %				4815	74·3 %
Missing				7	0·1 %				22	0·5 %				0	0·0 %
Urban/Rural location															
Urban				6290	48·4 %				1740	41·0 %				1647	25·4 %
Rural				6709	51·6 %				2508	59·0 %				4830	74·6 %
Missing				0	0·0 %				0	0·0 %				0	0·0 %
Years of education															
Missing	5·47	4·52	0–23	178	1·4 %	4·10	5·32	0–26	64	1·5 %	3·64	4·72	0–23	19	0·3 %
Currently employed				1771	13·6 %				90	2·1 %				1929	29·8 %
Never worked/homemaker				5060	38·9 %				526	12·4 %				880	13·6 %
Retired				885	6·8 %				668	15·7 %				1034	16·0 %
Seeking work/Not employed				4980	38·3 %				2949	69·4 %				2633	40·7 %
Employed				303	2·3 %				15	0·4 %				1	0·02 %
Missing															
Smoker															
Never				8505	65·4 %				3130	73·7 %				3057	47·2 %
Previous				774	6·0 %				567	13·4 %				339	5·2 %
Current				3457	26·6 %				525	12·4 %				3079	47·5 %
Missing				263	2·0 %				26	0·6 %				2	0·03 %
Alcohol consumption															
Don’t drink				8811	67·8 %				1753	41·3 %				5447	84·1 %
≤ 2 drinks less than once/week				1070	8·2 %				799	18·8 %				312	4·8 %
≤ 2 drinks at least once/week				851	6·6 %				625	14·7 %				179	2·8 %
> 2 drinks less than once/week				253	2·0 %				57	1·3 %				80	1·2 %
> 2 drinks at least once/week				951	7·3 %				218	5·1 %				84	1·3 %
Missing				1063	8·2 %				796	18·7 %				375	5·8 %
BMI (kg/m^2^)															
Missing	23·74	3·43	13·30–38·06	852	6·6 %	22·95	4·90	13·16–42·08	136	3·2 %	20·50	4·06	11·15–37·15	192	3·0 %
Physical activity (min/week)															
Missing	670·07	720·62	0–5880	334	2·6 %	1092·7	824·83	0–4140	117	2·8 %	1029·45	1039·76	0–5400	205	3·2 %
Sedentary behaviour (min/week)	1571·72	939·61	0–6720	545	4·2 %	1545·49	976·03	0–6720	62	1·5 %	1389·85	1081·58	0–6720	15	0·2 %

	Mexico (*n* 2016)					Russia (*n* 2795)					South Africa (*n* 3752)				
Variable	Mean	sd	Range	*n*	%	Mean	sd	Range	*n*	%	Mean	sd	Range	*n*	%
Successful ageing															
No				1847	91·6 %				2475	88·6 %				3342	89·1 %
Yes				82	4·1 %				242	8·7 %				243	6·5 %
Missing				87	4·3 %				78	2·8 %				167	4·5 %
Successful ageing score															
Missing^*^	2·74	1·55	0–8	489	24·3 %	2·58	1·73	0–8	496	17·7 %	2·19	1·44	0–8	948	25·3 %
Servings of fruits consumed on typical day															
Missing	1·6	1·0	0–5	153	7·6 %	1·5	1·0	0–5	382	13·7 %	1·6	1·1	0–5	250	6·7 %
Servings of vegetables consumed on typical day															
Missing	1·65	1·00	0–5	152	7·5 %	1·79	0·99	0–5	417	14·9 %	1·94	1·11	0–6	249	6·6 %
Servings of fruits and vegetables consumed on a typical day	3·18	1·65	0–10	195	9·7 %	3·21	1·68	0–10	501	17·9 %	3·52	1·94	0–11	291	7·8 %
Age (years)	69·01	9·04	50–105			64·34	9·91	50–100			62·65	9·68	50–113		
Gender															
Male				785	38·9 %				1004	35·9 %				1597	42·6 %
Female				1231	61·1 %				1791	64·1 %				2155	57·4 %
Ethnicity^a^				–					*Russian:* 2069	74·0 %				*African/Black:* 2021	53·9 %
									*Caucasus:* 320	11·5 %				*White:* 260	6·9 %
									*Other:* 234	8·4 %				*Coloured:* 630	16·8 %
														*Indian/Asian:* 303	8·1 %
Missing									172	6·2 %				538	14·3 %
Marital status															
No partner				769	38·1 %				1150	41·1 %				1725	46·0 %
Partner				1162	57·6 %				1637	58·6 %				1956	52·1 %
Missing				85	4·2 %				8	0·3 %				71	1·9 %
Urban/Rural location															
Urban				1450	71·9 %				2085	74·6 %				2494	66·5 %
Rural				566	28·1 %				710	25·4 %				1258	33·5 %
Missing				0	0·0 %				0	0·0 %				0	0·0 %
Years of education															
Missing	4·36	4·09	0–25	120	6·0 %	11·08	3·81	0–25	53	1·9 %	5·74	4·75	0–26	623	16·6 %
Currently employed				1045	51·8 %				59	2·1 %				668	17·8 %
Never worked/homemaker				180	8·9 %				1421	50·8 %				1215	32·4 %
Retired				221	11·0 %				331	11·8 %				790	21·1 %
Seeking work/Not employed				485	24·1 %				966	34·6 %				978	26·1 %
Employed				85	4·2 %				18	0·6 %				101	2·7 %
Missing															
Smoker															
Never				1211	60·1 %				1929	69·0 %				2295	61·2 %
Previous				374	18·6 %				330	11·8 %				325	8·7 %
Current				346	17·2 %				501	17·9 %				938	25·0 %
Missing				85	4·2 %				35	1·3 %				194	5·2 %
Alcohol consumption															
Don’t drink				1013	50·3 %				703	25·2 %				2591	69·1 %
≤ 2 drinks less than once/week				182	9·0 %				869	31·1 %				259	6·9 %
≤ 2 drinks at least once/week				29	1·4 %				73	2·6 %				104	2·8 %
> 2 drinks less than once/week				170	8·4 %				342	12·2 %				149	4·0 %
> 2 drinks at least once/week				28	1·4 %				76	2·7 %				132	3·5 %
Missing				594	29·5 %				732	26·2 %				517	13·8 %
BMI (kg/m^2^)															
Missing	28·00	4·85	15·05–43·66	258	12·8 %	28·76	5·01	16·04–47·30	138	4·9 %	29·65	7·88	12·87–65·00	233	6·2 %
Physical activity (min/week)															
Missing	556·41	856·99	0–4380	98	4·86 %	1283·46	1028·52	0–5580	57	2·0 %	466·48	755·01	0–5040	270	7·2 %
Sedentary behaviour (min/week)	1069·77	934·22	0–5880	93	4·6 %	1954·83	1186·96	0–6720	144	5·2 %	1316·68	921·54	0–6720	182	4·9 %

a Ethnicity not reported for the majority of participants in Mexico.

Survey-weighted country-specific log-binomial and Poisson regression models were fitted to examine unadjusted and adjusted associations between each of fruit, vegetables, and fruit and vegetables combined and (i) successful ageing (no/yes) and (ii) successful ageing score, respectively. Log-binomial regression models were fitted as they provide estimates of prevalence ratios (PR) when modelling common outcomes which are more easily interpreted than odds ratios.

A complete case analysis was conducted under the assumption that the data were Missing Completely At Random (MCAR). This resulted in a sample of 8764 (67·4 % of eligible sample) for China, 2748 (64·7 %) for Ghana, 5182 (80·0 %) for India, 961 (47·7 %) for Mexico, 1244 (44·5 %) for Russia and 1917 (51·1 %) for South Africa. The MCAR assumption was assessed by comparing descriptive characteristics for the complete case sample to those omitted to assess whether the sample characteristics were similar (see online Supplementary Table 3). There were no notable differences in the fruit and vegetable intake variables, nor successful ageing (aside from China where 18·3 % were successfully ageing in the complete case sample compared to 9·2 % in the omitted sample) or the key confounders considered in the primary analysis. There were apparent differences in some covariates included in secondary analysis (e.g. physical activity and sedentary behaviour) across the countries meaning the assumption the data were MCAR may be questionable for these covariates. As these variables were only considered in sensitivity analyses, no further explorations of the missing data assumptions were conducted.

## Results

The percentage of participants successfully ageing ranged from 4·1 % in Mexico to 14·8 % in China (Table 1). China also had the lowest mean successful ageing score (indicating successful ageing in more components) at 1·7 (sd = 1·3), while Mexico had the highest at 2·7 (sd = 1·6). The average vegetable intake was less than two portions of vegetables per d for five of the six countries, with the mean ranging from 1·7 (sd = 1·0) portions per d in Mexico to 6·9 (sd = 3·9) portions per d in China (online Supplementary Table 3). Average fruit intake ranged from 0·9 (sd = 0·9) in India to 2·4 (sd = 2·2) in China. Combined average fruit and vegetable intake ranged from a low of 2·8 (sd = 1·4) for India to a high of 9·3 (sd = 4·7) for China.

**Figure 1.**
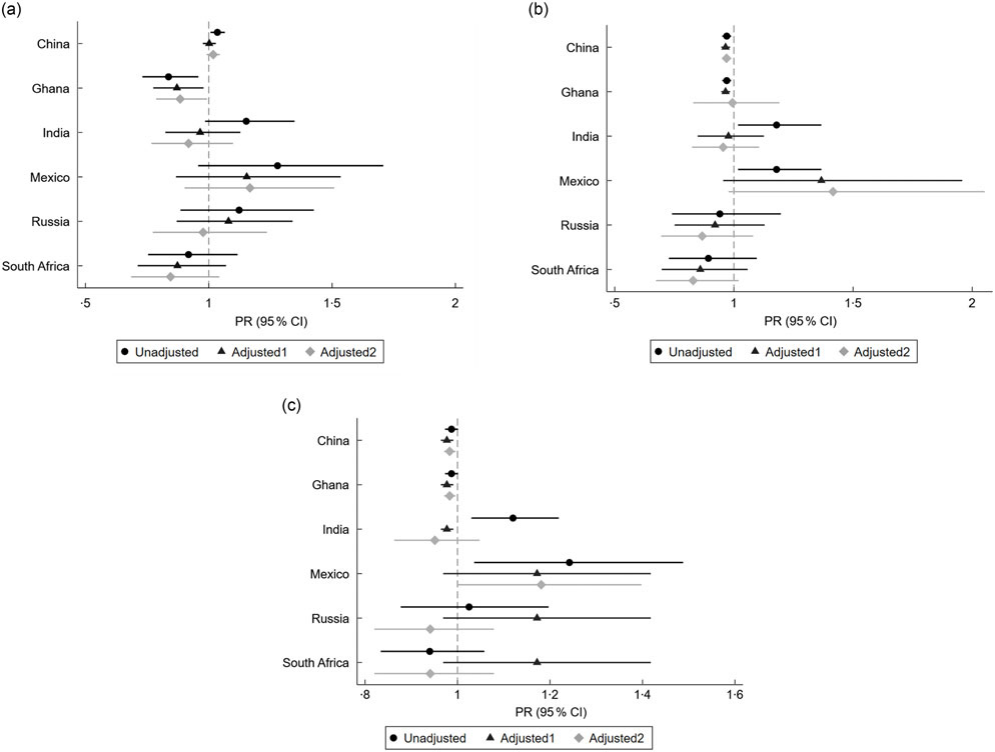
(a) Prevalence ratio (PR) of successful ageing by fruit intake. *Note: 0: not successfully ageing; 1 = successfully ageing. Adjusted model 1 includes age, sex, marital status, education, employment status, ethnicity (apart from Mexico where ethnicity was not reported by most participants) and urban/rural location which were identified as the minimal sufficient adjustment set to account for confounders. Adjusted model 2 includes model 1 + smoking status, alcohol intake, BMI, total physical activity and sedentary time. (b) PR of successful ageing by vegetable intake. * Note: 0: not successfully ageing; 1 = successfully ageing. Adjusted model 1 includes age, sex, marital status, education, employment status, ethnicity (apart from Mexico where ethnicity was not reported by most participants) and urban/rural location which were identified as the minimal sufficient adjustment set to account for confounders. Adjusted model 2 includes model 1 + smoking status, alcohol intake, BMI, total physical activity and sedentary time. (c) PR of successful ageing by fruit and vegetable intake. * Note: 0: not successfully ageing; 1 = successfully ageing. Adjusted model 1 includes age, sex, marital status, education, employment status, ethnicity (apart from Mexico where ethnicity was not reported by most participants) and urban/rural location which were identified as the minimal sufficient adjustment set to account for confounders. Adjusted model 2 includes model 1 + smoking status, alcohol intake, BMI, total physical activity and sedentary time.

Regression results indicated that whilst successful ageing may be higher for those who consumed more fruit in China, India and Mexico, where CI largely included values higher than 1 in unadjusted analyses, estimated PR attenuated after confounder adjustment. Successful ageing was lower for those with higher fruit consumption in Ghana, irrespective of variable adjustment (Fig. 1(a)), with an estimated PR of 0·87 (95 % CI 0·78, 0·98) in the primary adjusted analysis (online Supplementary Table 4). Considering vegetable intake (Fig. 1(b)), the results suggested that successful ageing was lower for those who consumed more vegetables in China (PR_adj1_: 0·97, 95 % CI 0·95, 0·98), with similar results obtained irrespective of confounder adjustment. In contrast, the results for Mexico suggest that successful ageing may be higher among those who consumed more vegetables (RR_adj1_: 1·38, 95 % CI 0·96, 1·96). The findings for combined fruit and vegetable intake were similarly mixed, with results mostly suggesting a positive effect of consumption on successful ageing in Mexico but a negative effect in China and Ghana (Fig. 1(c)).

**Figure 2.**
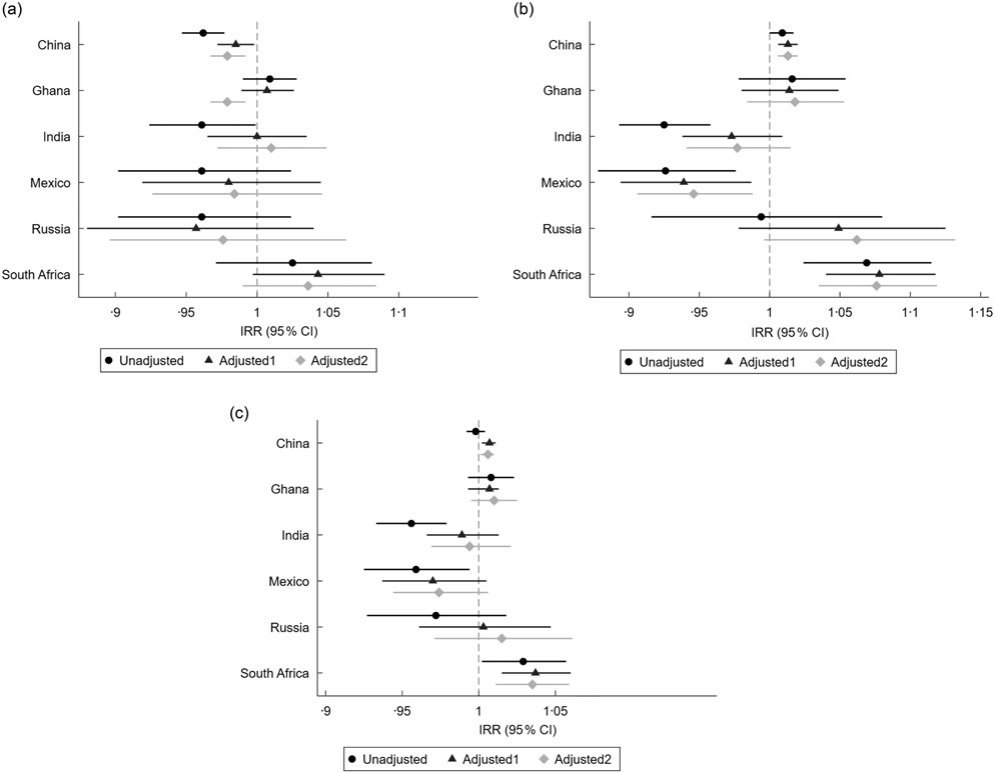
(a) Incidence rate ratio (IRR) of successful ageing score by fruit intake. * Note: Higher score indicates poorer outcomes. Adjusted model 1 includes age, sex, marital status, education, employment status, ethnicity (apart from Mexico where ethnicity was not reported by most participants) and urban/rural location which were identified as the minimal sufficient adjustment set to account for confounders. Adjusted model 2 includes model 1 + smoking status, alcohol intake, BMI, total physical activity and sedentary time. (b) IRR of successful ageing score by vegetable intake* Note: Higher score indicates poorer outcomes. Adjusted model 1 includes age, sex, marital status, education, employment status, ethnicity (apart from Mexico where ethnicity was not reported by most participants) and urban/rural location which were identified as the minimal sufficient adjustment set to account for confounders. Adjusted model 2 includes model 1 + smoking status, alcohol intake, BMI, total physical activity and sedentary time. (c) IRR of successful ageing score by fruit and vegetable intake. * Note: Higher score indicates poorer outcomes. Adjusted model 1 includes age, sex, marital status, education, employment status, ethnicity (apart from Mexico where ethnicity was not reported by most participants) and urban/rural location which were identified as the minimal sufficient adjustment set to account for confounders. Adjusted model 2 includes model 1 + smoking status, alcohol intake, BMI, total physical activity and sedentary time.

Findings were also conflicted when examining successful ageing score (online Supplementary Table 5). In China, on average those who consumed more fruit had lower (i.e. better) successful ageing scores (Fig. 2(a)), while those who consumed more vegetables (Fig. 2(b)) had higher (poorer) successful ageing scores. The latter finding was also observed in South Africa. Findings for Mexico were also in agreement with prior results, with lower (better) scores among those with higher vegetable intake. Findings for combined fruit and vegetable intake were similar with higher fruit and vegetable intake associated with higher (poorer) successful ageing scores in China and South Africa (Fig. 2(c)).

## Discussion

The aim of this study was to develop successful ageing profiles and examine associations between fruit and vegetable intake and successful ageing across six LMIC (China, Ghana, India, Mexico, Russia and South Africa). The prevalence of successful ageing ranged from 4 % in Mexico to 15 % in China. Overall associations between fruit and vegetable intake were limited and varied substantially across countries, suggesting the need for a more detailed assessment of dietary intake in understanding country- or region-specific influences on successful ageing.

There was a large variation by country in the proportion of participants reported to have successfully aged, which may be explained by variation in health behaviours and disease incidence. China, which reported the highest prevalence of successful ageing reported lower levels of chronic disease, higher levels of physical function (walking speed and grip strength) and higher levels of good mental health compared to countries with lower successful ageing proportions. In contrast, Mexico reported higher disability levels, poorer physical function (grip strength) and higher levels of chronic disease, especially diabetes.

The observed differences in chronic disease and function by region are reflective of other global analyses^(22,23)^. A recent study of 4019 Chinese older adults also reported rates of successful ageing of 19 % for men and 10 % for women, similar to the rate of 15 % reported in this study^(24)^. In contrast, previous studies in Mexico have reported higher rates of successful ageing, ranging from 10 %^(25)^ to 13 %^(26)^. However, their successful ageing definition, informed by Rowe and Kahn^(27)^, differed to that in the current study and included less health-based items and a social engagement item.

Similarly, a more recent study in India of 21 343 community-dwelling individuals aged 65 years and over from the Longitudinal Ageing Study in India (LASI) Wave 1 in 2017–2018 reported higher successful ageing rates of 27·2 % compared to 6 % in the current study^(28)^. This variation in successful ageing rates can be partially explained by differences in disease rates across the samples, where 83·3 % reported no major diseases in the LASI study compared to only 59·7 % in the current study. The current study used both formal diagnosis of chronic conditions by a healthcare professional and a proxy self-report of symptoms to indicate presence of chronic disease, which is designed for individuals with low health literacy or low access to healthcare, compared to the more formal diagnosis of disease criteria used in LASI. This may have increased chronic disease rates recorded and therefore reduced rates of successful ageing in our sample compared to other studies and points to variation in successful ageing rates dependant on the scoring approach and items included. Together these findings suggest there may be a need to consider patterns of successful ageing which are country-specific as one definition of successful ageing may not be relevant to all regions and all older adults.

The current study shows mixed associations between fruit and vegetable intake and successful ageing, varying across the LMIC considered. In China, and to a limited extent South Africa, negative associations with vegetable intake and successful ageing were observed. The limited and contradictory associations contrast those from Western Europe, Australia and the USA, where more consistent associations have been observed between fruit and vegetable intake and reduced disease burden^(29)^.

Although research into fruit and vegetable intake and health outcomes in LMIC is limited, a previous study also conducted using SAGE data reported regional variations in relationships between lifestyle risk factors and CVD^(30)^. Results showed obesity was associated with angina in China and Russia, whilst smoking was a stronger risk factor in India and South Africa. Insufficient fruit and vegetable intake was also explored and was only associated with angina risk in Ghana. This contrasts findings in the current study where higher fruit intake was associated with lower successful ageing in Ghana. A previous study of 17 700 community-dwelling dementia-free Chinese adults aged 65 years and over living in Hong Kong found that participants who met WHO recommendations of at least three serves of vegetables and two serves of fruit per d had a reduced risk of dementia after 6 years^(31)^. In the China cohort of the current study, associations were mixed, with higher fruit intake associated with higher successful ageing, whilst higher vegetable intake was associated with lower successful ageing.

These inconsistent and contradictory findings warrant further consideration. A key consideration is the role of economic development and associated nutrition transition. Economic development can lead to improved socio-economic factors, healthcare access and increased longevity of older people. However, increased exposure to a Western diet at the same time leads to increases in obesity and associated chronic disease detrimental to successful ageing^(32)^. These competing factors can create a paradox of increasing socio-economic status, poorer health outcomes and contradictory findings across countries depended on the time, rate and stage of transition experienced. For example, a systematic assessment of dietary patterns across 187 countries from 1990 to 2010 showed diets were heterogenous across regions. Increases in both healthy dietary items, including fruits and vegetables, and unhealthy dietary items, such as sugar-sweetened beverages and processed foods, were observed in middle-income countries, reflecting increased access to both healthy and unhealthy foods^(33)^. At the same time, lower-income countries reported increases in unhealthy food items and no improvements in consumption of healthy food items. Overall, global consumption of healthy foods increased modestly over time, but any improvement was shadowed by a greater increase in unhealthy foods^(33)^. Therefore, the dietary changes and associated health outcomes are complex and more studies looking at multiple aspects of both healthy and unhealthy dietary intakes are needed to further understand the relationship between diet and successful ageing.

The current study includes countries from a range of economic conditions with different levels of nutrition transition, which may have contributed to the contrasting findings. The countries included can be classified by the development level according to the UN Human Development Index (HDI) from 2007 according to two groups: higher HDI (Russia, China and Mexico) and lower HDI (South Africa, India and Ghana) to see if there are similar patterns of associations between fruit and vegetable intake and successful ageing observed across countries with similar economic development levels^(34)^. However, no consistent patterns within higher and lower HDI country groups were found. Therefore, the variations across countries are not specifically explained solely by socio-economic differences between countries, but it is likely a range of complex socio-economic and cultural differences may be at play instead. This highlights the importance of conducting studies on ageing across countries with economic and cultural differences as applying learning from studies conducted in Western or high-income countries as a one-size-fits-all approach will not be relevant.

Economic development and nutrition transition can also occur at different rates within a country, with rural areas often lagging behind urban city centres leading to regional variations within countries. A comparison of associations between the Mediterranean diet components and successful ageing across 1128 city-based and 2221 island-based participants aged 50 years and over in Greece showed marked variations^(35)^. Specifically, consumption of legumes, vegetables, cereals and poultry were associated with successful ageing among the islanders, but not the city-based participants^(35)^. It is thus likely that determinants of successful ageing are varied and regional-specific, highlighting the need for further investigation in LMIC.

While fruits and vegetables have often been considered key components of overall diet quality^(5)^, on their own they may not be adequate markers of the relevant aspects of diet quality for successful ageing in LMIC or those going through nutrition transition. A range of broader factors including healthy and unhealthy dietary patterns, macro and micronutrients and ultra-processed food consumption may be a better marker of diet quality. The recent PURE study examined dietary intakes across eighteen low- and middle- and high-income countries and found that diets high in fruits, legumes and vegetables were associated with lower mortality and also that diets high in carbohydrate and low in protein were associated with increased mortality^(36,37)^. In addition, this study found dietary patterns were markedly different compared to previous studies in Western populations, with most participants from low-income and middle-income countries consuming a very high carbohydrate diet (at least 60 % of energy) mainly from refined sources such as white rice and white bread^(37)^.

Therefore, it is possible that intake of individual food items or macronutrients may reflect markedly different dietary patterns and influences of socio-economic status across countries, and broader investigation of dietary intake is needed to understand associations between diet and health further in LMIC. It may be that in some LMIC, higher vegetable intake is a marker of traditional diet in rural areas or lower socio-economic regions where healthcare access is limited compared to urban areas^(38)^. These areas could be characterised by low intake of meat and fruit and high intakes of grains and vegetables^(38,39)^. Furthermore, the cultural context in which these foods are prepared and consumed including cultivation methods, transport and storage, preparation and cooking methods which could all contribute to nutrient content and availability and were not able to be assessed in the current study. Another important consideration is if the fruit and vegetables are grown in lower socio-economic areas which are close to industrial areas, irrigated with waste water or pesticide pollution, this could increase the accumulation of heavy metals or other contaminants^(40–42)^. Consumption of these contaminated foods has the potential to cause serious health problems and contribute to unexpected associations with health outcomes.

Another important consideration is the types of fruits and vegetables consumed was not assessed and would influence the nutrient intake and potential health benefits. Evidence from the nation-wide NSSO Household Consumer Expenditure Survey in India showed fruit and vegetable intake amounts were similar among urban and rural households, but a significant portion of intake was from energy-dense options such as potatoes and bananas^(43)^. A greater diversity in types of vegetables consumed was observed in higher income households, but the same relationship was not observed with fruit types. Similar findings have been previously observed in EPIC study, with British older adults with low socio-economic position (SEP) reporting lower fruit and vegetable variety but not lower levels of intake^(44)^. A recent umbrella review reported benefits of fruit and vegetable variety on body weight, lipid profile, inflammation, chronic disease and mortality, suggesting fruit and vegetable variety may have benefits independent to overall intake^(45)^.

SEP is known to be an important determinant of health, and variations in economic transition may be contributing to conflicting findings across the studies. Different relationships between SEP and health have been observed within SAGE previously, specifically higher household income was associated with reduced odds of angina in China, India and Russia, whilst the opposite was observed in South Africa^(30)^. The current study adjusted for markers of SEP in the form of education, employment status and ethnicity; however, it is possible that some residual confounding occurred and the influence of SEP on the associations between fruit and vegetable intake and successful ageing in the current study is unknown.

### Strengths and limitations

This study has a number of strengths. First, the analysis across the six LMIC was conducted using SAGE, a study with consistent survey design, recruitment and data collection methods across countries, which allows for greater comparison across the countries compared to individual studies in each region. SAGE used measures similar to previous large cohort studies of older populations in high-income countries, which allows for more meaningful comparison of results^(13)^. The assessment of successful ageing was conducted using a multidimensional measure, which incorporated aspects of physical and mental health, function and disability allowing for a more comprehensive assessment compared to the use of single measures. The regression analysis considered causal pathways through directed acyclic graphs to determine relevant covariates, which is recommended practice in epidemiology^(46)^. Population weights were applied to the regression analysis, which increases the representativeness of the sample and allow more accurate estimates to be drawn.

However, there are limitations to acknowledge. First, this was a cross-sectional analysis and therefore conclusions regarding the direction of causality in the relationships observed cannot be drawn. Follow-up surveys have been conducted, with release of data planned, which will allow longitudinal analyses^(13)^. Also, previous reports have indicated the interview duration was long (mean time for Wave 1 was 2·5 h), and the resulting burden on interviewers and respondents may have implications for data quality and may have contributed to the levels of missing data observed^(13)^. In addition, some of the items in the successful ageing score relied on self-reported data rather than objective measures, although this has also been the case in successful ageing scores previously^(14)^. Some variables examined (e.g. BMI and physical activity) included some extreme values which make these observations questionable. Given questions around the accuracy of these variables, in addition to these variables not identified as key confounders in our directed acyclic graphs, these were only considered in sensitivity analyses in the secondary adjustment models. Although some values were questionable, adjustment for these variables did not result in major changes to the estimated effects of fruit and vegetable intake on successful ageing. Finally, although SAGE included detailed assessment of health and ageing outcomes, the assessment of dietary intake was limited, with only information on fruit and vegetable intake and alcohol consumption available. These limited dietary intake variables did not allow for exclusion of individuals with implausible energy intakes or adjustment of energy intake in the analysis. However, to partially address this limitation, we were able to include key determinants of energy intake in the adjusted regression models through the inclusion of age, sex, BMI, physical activity and sedentary time. Further exploration of these associations using more detailed dietary data may help to unpack the differences in associations observed in this study.

### Implications and future research

These initial findings suggest the association between fruit and vegetable intake and successful ageing vary widely across LMIC and compared to Western populations more commonly studied in this field. There is a strong need for further investigation of dietary intake and successful ageing in these regions, including more detailed dietary intake assessment to complement the multicomponent examination of health and ageing outcomes completed in the current study. This is supported by a previous study of individual and combined health behaviours including fruit and vegetable intake which found moderate associations with individual behaviours but more strong associations with a combined overall score^(15)^. Future investigations should also consider longitudinal data to determine how this relationship changes over time. Finally, investigation of socioecological determinants of dietary intake in older age are vital to understand regional and cultural-specific influences on food. It is likely that the exact dietary components required to support successful ageing may vary by country. Further research to understand what these specific components are and how they relate to socio-economic conditions and cultural contexts of older people needs further consideration to determine the most effective dietary intervention methods to support successful ageing.

### Conclusion

Associations between fruit and vegetable intake and successful ageing in LMIC are inconsistent, and the influences on successful ageing may vary by country. Interventions to meet the challenges of the ageing population will need to be carefully designed to consider influences on successful ageing in individual countries.

## Supporting information

Milte et al. supplementary material 1 Milte et al. supplementary material

Milte et al. supplementary material 2 Milte et al. supplementary material

## Supplementary material

For supplementary material accompanying this paper visit https://doi.org/10.1017/S1368980024001976
