# Supplementary material for: Cross-sectional associations between fruit and vegetable intake and successful ageing across six countries: findings from the WHO Study on global AGEing and adult health (SAGE)
Source: Public Health Nutr. 2024 Oct 21;27(1):e244. doi: 10.1017/S1368980024001976 (PMC11705009; doi:10.1017/S1368980024001976)
Supplement: Milte et al. supplementary material 1 — Milte et al. supplementary material [file S1368980024001976sup001.docx]

**Successful ageing score details**

*History of chronic disease*

History of chronic disease was measured using self-reported prior diagnosis of the following diseases: stroke, angina and diabetes. In addition, four self-report items relating to whether or not the participants had ever suffered from paralysis or loss of feeling, or pain in chest normally or if exerting during the last 12 months were included. As some participants were from remote or rural areas where access to health care may be limited, restricting formal diagnoses of certain conditions, these outcomes were included as proxy measures of diagnoses of stroke or angina, respectively.

*Physical function*

Physical function was objectively measured using both a timed walk at a normal pace and objectively measured grip strength using a dynamometer. These capture different aspects of physical capacity (namely locomotion and strength) and were considered separately when scoring successful ageing [1].

*Timed walk*

For the timed walk, participants were asked to walk 4 metres over a flat and straight surface which was free of obstacles, using a cane or walking aid if necessary, at their usual pace and the time taken was recorded. Age and sex-specific cut-points for slow gait were used to indicate poor physical function, based on cut-points from Oh-Park et al. 2010 [2] (Supplementary Table 1). Those who were unable to walk and could not complete the walk at the usual pace were defined as having poor walking function.

*Grip strength*

Before assessing grip strength, participants were first asked if they had had surgery on either arm, hand or wrist in the last 3 months or if they had arthritis or pain in either hand or wrist. If a participant responded yes for either hand, grip strength was not assessed for that hand. A dynamometer was used to assess grip strength, with two measurements taken for each hand. Since most participants had data available for both hands, the average of all attempts for both hands were used. If measurements for only one hand was available (e.g. due to surgery on the other hand), only the average of measurements for this hand was used. According to the European Working Group on Sarcopenia, grip strengths of < 30 kg for men and < 20 kg for women are classed as poor grip strength [3, 4]. These cut-points were used in this analysis to define poor physical function according to grip strength.

*Respiratory function*

Respiratory function was measured based on responses to three questions relating to self-report of chronic lung disease, shortness of breath and coughing or wheezing (described in Supplementary Table 1). Participants who responded no to all of these questions were classified as having good respiratory function. Those who responded yes to any of the three questions were classified as having poor respiratory function for the purposes of this study.

*Cardiovascular function*

Cardiovascular function was determined using objectively measured systolic blood pressure (SBP) and diastolic blood pressure (DBP). Up to three measurements were taken for each participant by the survey interviewer with the average calculated for each individual. Participants were defined to have good cardiovascular function if they did not meet the WHO threshold for having high blood pressure (i.e. SBP < 140 mmHg and DBP < 90 mmHg) [5]. Those who had either SBP ≥ 140 mmHg or DBP ≥ 90 mmHg (or both) were classed as having poor cardiovascular function.

*Cognitive function*

Cognitive function was assessed using interviewer administered verbal recall tests, as well as self-report of difficulties with concentration or memory during the last 12 months. In the verbal recall test, participants were given a list of 10 words to test their memory. Participants were then asked to recall the list of words, in no particular order three times immediately after the list was provided, followed by an attempt to recall the list a fourth time after a 10 minute delay during which other tests were completed. The number of words correctly recalled at each test was summed. Participants were defined as having good verbal recall if they scored above or equal to the country-specific 10^th^ percentile (see Supplementary Table 1 for details) and as poor verbal recall if below this value.

Participants were also asked to report how much difficulty they had with concentrating or remembering things over the last 30 days. Responses were provided on a 5-point scale (none, mild, moderate, severe, extreme/cannot do). Participants who responded severe or extreme on this scale were classified as having poor concentration or memory while others were classified as having good concentration or memory.

Participants were defined as having good cognitive function if they had both good verbal recall and good concentration or memory and as having poor cognitive function otherwise.

*Disability*

Participants responded to questions from the 12-item WHO Disability Assessment Schedule (WHODAS), in addition to a further 14 questions relating to activities of daily living (ADL) or instrumental activities of daily living (IADL). Each question was ranked on a 5-point scale (none, mild, moderate, severe, extreme/cannot do), with an additional option of not applicable. The presence of disability was defined as reporting either severe or extreme difficulty in one or more of the items from either the WHODAS, ADL or IADL.

*Mental health*

Participants reported whether or not they had ever been diagnosed with depression. In addition, participants completed a series of questions about symptoms related to the diagnosis of mild, moderate or severe depression over the last 12 months based on the International Classification of Disease 10^th^ Revision, Diagnostic Criteria for Research (ICD-10-DCR) [6]. This was used to quantify depression symptoms. Participants were defined as having sufficient symptoms for diagnosis of mild, moderate or severe depression if they met the ICD-10-DCR criteria as previous described [7].

Research has indicated the depression may be underdiagnosed in older adults in several WHO SAGE countries including India, Ghana and China [8]. Therefore, the presence of mental health difficulties was defined as either reporting diagnosis with depression or having sufficient symptoms for diagnosis of mild, moderate or severe depression according to the ICD-10-DCR [7]. Participants were reported to have good mental health if they did not report diagnosis with depression and did not have sufficient symptoms for diagnosis of mild, moderate or severe depression.

*Successful ageing*

As defined elsewhere, [9] participants were defined to be successfully ageing if they had no history of chronic disease, had good walking function, good grip strength, good respiratory function, good cardiovascular function, good cognitive function, no disability and good mental health. Participants were not successfully ageing if they had poor outcomes for at least one of these components. In addition to the binary outcome, each of the eight components was scored in a successful ageing score, as described in Supplementary Table 1. Lower scores indicate more successful ageing.

## **Potential confounders**

Age, gender, years of schooling completed (including higher education), marital status (never married/currently married or cohabiting/separated or divorced/widowed), employment (never worked/retired/seeking employment/currently employed), location (urban/rural) and country-specific ethnicities were considered as potential confounders (see Supplementary Figure 1). Ethnic groups varied by country and were defined as follows: China (Han/other); South Africa (African Black/White/Coloured/Indian or Asian); Ghana (Akan/Ga-Adangbe/Ewe/Gruma/Mole-Dagbon/other); India (scheduled tribe or class/no caste or tribe/other); and Russian Federation (Russian/Caucasus/other). Ethnicity was not considered for Mexico as most participant recorded “none” or “missing”. Urban/rural location was not included as it was not required for minimal adjustment. All other potential confounders were adjusted for in the primary adjusted analyses (adjustment 1).

In sensitivity analyses, total minutes of physical activity per week and total minutes of sedentary time per week (derived from the Global Physical Activity Questionnaire [GPAQ]) [10], smoking status (never smoked/former smoker/current smoker), alcohol consumption over the past 12 months (never drink/≤2 drinks less than once per week/≤2 drinks more than once per week/>2 drinks less than once per week/>2 drinks more than once per week), and body mass index (kg/m^2^; derived from interviewer measurements of height and weight) were included in the models (adjustment 2). In the absence of clear guidance on harmful alcohol consumption across the WHO SAGE countries, the cut-point of 2 alcoholic drinks was chosen based on the Australian National Health and Medical Research Council risk guidelines for lifetime risk of harm from alcohol-related disease or injury. These variables were only considered in secondary adjustment as although all are likely to predict successful ageing, the relationship between these variables and fruit and vegetable intake is unclear (see Supplementary Figures 1 and 2).

**Supplementary Table 1:** Defining successful ageing score

| **Component** | **Question(s)/Measurement** | **Score** |
| --- | --- | --- |
| History of chronic disease | 1. Have you ever been told by a health professional that you have had a stroke?  2. Have you ever been diagnosed with angina or angina pectoris (a heart disease)?  3. Have you ever been diagnosed with diabetes (high blood sugar)?  4. Have you ever suffered from sudden onset of paralysis or weakness in your arms or legs on one side of your body for more than 24 hours?  5. Have you ever had, for more than 24 hours, sudden onset of loss of feeling on one side of your body without anything having happened to you immediately before?  6. During the last 12 months, have you experienced any pain or discomfort in your chest when you walk uphill or hurry?  7. During the last 12 months, have you experienced any pain or discomfort in your chest when you walk at an ordinary pace on level ground? | Any chronic disease  No: 0  Yes: 1 |
| Walking physical function | Timed 4m walk at normal pace above cut-points described below.  Cut-points for good walking function:  Males  Age ≥ 50 - < 55yrs: ≥ 0.703 m/s  Age ≥ 55 - < 60yrs: ≥ 0.690 m/s  Age ≥ 60 - < 65yrs: ≥ 0.667 m/s  Age ≥ 65 - < 70yrs: ≥ 0.616 m/s  Age ≥ 70 - < 75yrs: ≥ 0.582 m/s  Age ≥ 75 - < 80yrs: ≥ 0.562 m/s  Age ≥ 80yrs : ≥ 0.468 m/s  Females  Age ≥ 50 - < 55yrs: ≥ 0.636 m/s  Age ≥ 55 - < 60yrs: ≥ 0.626 m/s  Age ≥ 60 - < 65yrs: ≥ 0.597 m/s  Age ≥ 65 - < 70yrs: ≥ 0.549 m/s  Age ≥ 70 - < 75yrs: ≥ 0.500 m/s  Age ≥ 75 - < 80yrs: ≥ 0.436 m/s  Age ≥ 80yrs : ≥ 0.361 m/s | Good walking physical function  No: 1  Yes: 0 |
| Grip strength physical function | Dynamometer measured grip strength (kg) ≥30 kg for males and ≥20 kg for females to be classed as good grip strength. | Good grip strength physical function  No: 1  Yes: 0 |
| Respiratory function | 1. Have you ever been diagnosed with chronic lung disease (emphysema, bronchitis, COPD)?  2. During the last 12 months, have you experienced any shortness of breath at rest? *(while awake)*  3. During the last 12 months, have you experienced any coughing or wheezing for ten minutes or more at a time? | Any respiratory function problems  No: 0  Yes: 1 |
| Cardiovascular function | Measured systolic and diastolic blood pressure.  Cut-points for good cardiovascular function:  SBP < 140 mmHg  DBP < 90 mmHg | Good cardiovascular function  No: 1  Yes: 0 |
| Cognitive function | 1) Interviewer recorded verbal recall (10 words, 4 tests) with country-specific cut-points based on 10^th^ percentile.  Good verbal recall definition  China: ≥13 words  Ghana: ≥15 words  India: ≥ 14 words  Mexico: ≥ 12 words  Russia: ≥ 15 words  South Africa: ≥ 15 words  2) Self-report difficulty with concentrating or remembering things over the last 30 days.  Good concentration/memory defined as responses of none, mild or moderate.  Good cognitive function if both good verbal recall and good concentration or memory. | Good cognitive function  No: 1  Yes: 0 |
| Disability | WHODAS and ADL/IADL items  Overall in the last 30 days, how much difficulty did you have…  1) …in learning a new task (for example, learning how to get to a new place, learning a new game, learning a new recipe)?  2) …with making new friendships or maintaining current friendships?  3) …with dealing with strangers?  4) …in sitting for long periods?  5) …in walking 100 meters?  6) …in standing up from sitting down?  7) …in standing for long periods?  8) …with climbing one flight of stairs without resting?  9) …with stooping, kneeling or crouching?  10) …picking up things with your fingers(such as picking up a coin from a table)?  11) …in taking care of your household responsibilities?  12) …in joining in community activities (for example, festivities, religious or other activities) in the same way as anyone else can?  13) …in extending your arms above shoulder level?  14) …concentrating on doing something for 10 minutes?  15) …in walking a long distance such as a kilometer?  16) …in bathing/washing your whole body?  17) …in getting dressed?  18) …in your day to day work?  19) …with carrying things?  20) …with moving around inside your home (such as walking across a room)?  21) …with eating (including cutting up your food)?  22) …with getting up from lying down?  23) …with getting to and using the toilet?  24) …with getting where you want to go, using private or public transport if needed?  25) …getting out of your home?  26) In the last 30 days, how much have you been emotionally affected by your health condition(s)?  Responses of none, mild, moderate for any of the above were coded as a good outcome for that item (extreme or severe were coded as poor). Absence of disability was defined as having a good outcome for all of the above. | Absence of disability  No: 1  Yes: 0 |
| Mental health | 1) Depression diagnosis:  Have you ever been diagnosed with depression?  2) ICD-10 depression symptoms  - During the last 12 months, have you had a period lasting several days when you felt sad, empty or depressed?  - During the last 12 months, have you had a period lasting several days when you lost interest in most things you usually enjoy such as personal relationships, work or hobbies/recreation?  - During the last 12 months, have you had a period lasting several days when you have been feeling your energy decreased or that you are tired all the time?  - Was this period [of sadness/loss of interest/low energy] for more than 2 weeks?  - Was this period [of sadness/loss of interest/low energy] most of the day, nearly every day?  - During this period, did you lose your appetite?  - Did you notice any slowing down in your thinking?  - Did you notice any problems falling asleep?  - Did you notice any problems waking up too early?  - During this period, did you have any difficulties concentrating; for example, listening to others, working, watching TV, listening to the radio?  - Did you notice any slowing down in your moving around?  - During this period, did you feel anxious and worried most days?  - During this period, were you so restless or jittery nearly every day that you paced up and down and couldn’t sit still?  - During this period, did you feel negative about yourself or like you had lost confidence?  - Did you frequently feel hopeless - that there was no way to improve things?  - During this period, did your interest in sex decrease?  - Did you think of death, or wish you were dead? | Good mental health  No: 1  Yes: 0 |

**Supplementary Table 2:** Descriptive characteristics of sub-components of successful ageing

|  | **CHINA**  **(N = 12999)** | **GHANA**  **(N = 4248)** | **INDIA**  **(N = 6477)** | **MEXICO**  **(N = 2016)** | **RUSSIA**  **(N = 2795)** | **SOUTH AFRICA**  **(N = 3752)** |
| --- | --- | --- | --- | --- | --- | --- |
| **History of chronic disease** |  |  |  |  |  |  |
| Any history of chronic disease, % |  |  |  |  |  |  |
| *No* | 72.8 | 72.5 | 59.7 | 61.3 | 48.4 | 72.3 |
| *Yes* | 22.4 | 27.0 | 40.2 | 34.5 | 50.1 | 23.0 |
| *Missing* | 4.8 | 0.5 | 0.1 | 4.2 | 1.5 | 4.8 |
|  |  |  |  |  |  |  |
| Stroke, % |  |  |  |  |  |  |
| *No* | 94.8 | 97.0 | 97.7 | 91.2 | 93.0 | 91.8 |
| *Yes* | 3.3 | 2.6 | 2.2 | 4.6 | 5.5 | 3.5 |
| *Missing* | 1.9 | 0.4 | 0.0 | 4.2 | 1.5 | 4.7 |
|  |  |  |  |  |  |  |
| Paralysis, % |  |  |  |  |  |  |
| *No* | 92.3 | 95.8 | 96.3 | 91.0 | 93.0 | 91.4 |
| *Yes* | 3.0 | 3.7 | 3.7 | 4.8 | 5.4 | 3.8 |
| *Missing* | 4.7 | 0.5 | 0.0 | 4.2 | 1.6 | 4.8 |
|  |  |  |  |  |  |  |
| Loss of feeling, % |  |  |  |  |  |  |
| *No* | 93.4 | 96.1 | 96.8 | 92.2 | 93.4 | 91.8 |
| *Yes* | 1.7 | 3.4 | 3.2 | 3.6 | 5.0 | 3.4 |
| *Missing* | 4.9 | 0.5 | 0.0 | 4.2 | 1.6 | 4.8 |
|  |  |  |  |  |  |  |
| Angina, % |  |  |  |  |  |  |
| *No* | 89.4 | 96.3 | 95.1 | 93.8 | 67.7 | 89.8 |
| *Yes* | 8.6 | 3.3 | 4.9 | 1.9 | 30.8 | 5.5 |
| *Missing* | 2.0 | 0.4 | 0.0 | 4.2 | 1.5 | 4.7 |
|  |  |  |  |  |  |  |
| Chest pain if exerting, % |  |  |  |  |  |  |
| *No* | 88.7 | 82.1 | 71.3 | 85.8 | 64.7 | 84.6 |
| *Yes* | 8.7 | 17.5 | 28.7 | 10.0 | 33.7 | 10.7 |
| *Missing* | 2.5 | 0.4 | 0.0 | 4.2 | 1.6 | 4.7 |
|  |  |  |  |  |  |  |
| Chest pain normally, % |  |  |  |  |  |  |
| *No* | 92.5 | 83.6 | 81.4 | 89.5 | 67.7 | 86.5 |
| *Yes* | 5.1 | 16.1 | 18.5 | 6.3 | 30.8 | 8.7 |
| *Missing* | 2.4 | 0.4 | 0.0 | 4.2 | 1.5 | 4.7 |
|  |  |  |  |  |  |  |
| Diabetes, % |  |  |  |  |  |  |
| *No* | 91.6 | 95.8 | 92.8 | 76.2 | 89.4 | 86.1 |
| *Yes* | 6.4 | 3.8 | 7.2 | 19.6 | 9.1 | 9.2 |
| *Missing* | 2.0 | 0.4 | 0.0 | 4.2 | 1.5 | 4.7 |
|  |  |  |  |  |  |  |
| **Physical function - walk** |  |  |  |  |  |  |
| Slow walking speed, % |  |  |  |  |  |  |
| *No* | 90.9 | 64.3 | 85.6 | 74.5 | 64.4 | 57.9 |
| *Yes/cannot walk* | 3.5 | 33.4 | 12.4 | 19.6 | 33.1 | 33.2 |
| *Missing* | 5.6 | 2.3 | 1.9 | 5.9 | 2.5 | 8.9 |
|  |  |  |  |  |  |  |
| Walking speed (m/s), mean (SD) | 0.97 (0.23) | 0.76 (0.39) | 0.83 (0.23) | 1.23 (2.13) | 0.71 (0.30) | 0.85 (0.81) |
| *Missing* | 6.0 | 4.0 | 3.2 | 12.9 | 3.6 | 10.3 |
|  |  |  |  |  |  |  |
| **Physical function - grip** |  |  |  |  |  |  |
| Poor grip strength, % |  |  |  |  |  |  |
| *No* | 54.2 | 57.0 | 28.8 | 27.1 | 67.6 | 62.6 |
| *Yes* | 38.2 | 38.8 | 68.4 | 58.0 | 25.6 | 24.0 |
| *Missing* | 7.5 | 4.2 | 2.8 | 14.8 | 6.8 | 13.4 |
|  |  |  |  |  |  |  |
| Average grip strength (kg), mean (SD) | 26.44 (11.51) | 26.65 (11.81) | 21.77 (9.24) | 20.54 (8.18) | 29.36 (12.08) | 36.22 (18.70) |
| *Missing* | 7.6 | 4.2 | 2.8 | 14.8 | 6.8 | 13.4 |
|  |  |  |  |  |  |  |
| **Respiratory function** |  |  |  |  |  |  |
| Good respiratory function  *No*  *Yes*  *Missing* | 13.0  84.6  2.4 | 4.5  95.1  0.5 | 19.6  80.3  0.03 | 18.1  77.7  4.2 | 29.8  68.7  1.5 | 7.7  87.6  4.7 |
| Chronic lung disease, % |  |  |  |  |  |  |
| *No* | 89.6 | 99.0 | 95.9 | 90.0 | 80.0 | 93.1 |
| *Yes* | 8.5 | 0.6 | 4.1 | 5.8 | 18.5 | 2.3 |
| *Missing* | 1.9 | 0.4 | 0.0 | 4.2 | 1.5 | 4.7 |
|  |  |  |  |  |  |  |
| Shortness of breath, % |  |  |  |  |  |  |
| *No* | 91.2 | 96.8 | 86.6 | 86.8 | 85.8 | 89.7 |
| *Yes* | 6.7 | 2.8 | 13.4 | 9.0 | 12.7 | 5.7 |
| *Missing* | 2.1 | 0.4 | 0.0 | 4.2 | 1.5 | 4.7 |
|  |  |  |  |  |  |  |
| Coughing or wheezing, % |  |  |  |  |  |  |
| *No* | 92.4 | 96.8 | 87.5 | 86.2 | 80.5 | 90.0 |
| *Yes* | 5.4 | 2.8 | 12.5 | 9.6 | 18.0 | 5.3 |
| *Missing* | 2.2 | 0.4 | 0.0 | 4.2 | 1.5 | 4.7 |
|  |  |  |  |  |  |  |
| **Cardiovascular function** |  |  |  |  |  |  |
| Good cardiovascular function  *No*  *Yes*  *Missing* | 52.5  42.4  5.1 | 55.6  43.6  0.8 | 30.1  69.0  1.0 | 55.5  36.8  7.7 | 58.6  40.2  1.2 | 69.4  27.8  2.8 |
|  |  |  |  |  |  |  |
| Average SBP (mmHg), mean (SD) | 142.08 (23.33) | 138.35 (25.32) | 124.69 (20.54) | 148.10 (24.94) | 142.66 (21.27) | 146.75 (25.65) |
| *Missing* | *5.1* | *0.8* | *1.0* | *7.7* | *1.2* | *2.8* |
|  |  |  |  |  |  |  |
| Average SBP ≥ 140 mmHg, % |  |  |  |  |  |  |
| *No* | 48.7 | 57.5 | 79.0 | 37.5 | 48.1 | 41.7 |
| *Yes* | 46.2 | 41.7 | 20.0 | 54.8 | 50.7 | 55.5 |
| *Missing* | 5.1 | 0.8 | 1.0 | 7.7 | 1.2 | 2.8 |
|  |  |  |  |  |  |  |
| Average DBP (mmHg), mean (SD) | 84.68 (13.17) | 91.32 (16.69) | 81.80 (13.82) | 79.37 (11.64) | 87.82 (12.72) | 96.97 (17.27) |
| *Missing* | 5.1 | 0.8 | 1.0 | 7.7 | 1.2 | 2.9 |
|  |  |  |  |  |  |  |
| Average DBP ≥ 90, % |  |  |  |  |  |  |
| *No* | 64.5 | 49.5 | 74.8 | 75.5 | 56.3 | 35.1 |
| *Yes* | 30.4 | 49.6 | 24.3 | 16.7 | 42.5 | 61.9 |
| *Missing* | 5.1 | 0.8 | 1.0 | 7.7 | 1.2 | 2.9 |
|  |  |  |  |  |  |  |
| **Cognitive function** |  |  |  |  |  |  |
| Good cognitive function, %  *No*  *Yes*  *Missing* | 10.5  84.6  4.9 | 14.8  84.4  0.8 | 19.5  79.7  0.8 | 11.0  81.9  7.1 | 12.0  84.1  3.9 | 14.5  80.5  5.0 |
|  |  |  |  |  |  |  |
|  |  |  |  |  |  |  |
| Verbal recall, mean (SD) | 21.78 (6.73) | 22.11 (5.92) | 20.64 (5.67) | 19.41 (6.19) | 23.36 (6.38) | 23.29 (6.53) |
| *Missing* | 4.9 | 0.8 | 0.9 | 6.0 | 3.4 | 4.9 |
|  |  |  |  |  |  |  |
| Difficulties with concentration, % |  |  |  |  |  |  |
| *No* | 95.7 | 92.6 | 87.4 | 92.5 | 93.7 | 91.4 |
| *Yes* | 2.5 | 7.2 | 12.5 | 3.3 | 5.4 | 6.6 |
| *Missing* | 1.7 | 0.1 | 0.0 | 4.2 | 0.9 | 2.1 |
|  |  |  |  |  |  |  |
| **Disability** |  |  |  |  |  |  |
| Absence of disability  *No*  *Yes*  *Missing* | 17.4  75.0  7.6 | 45.4  51.8  2.8 | 58.7  37.9  3.4 | 44.8  44.3  10.9 | 33.6  58.4  8.0 | 33.9  58.1  8.0 |
| Difficulty learning a new task in last 30 days, % |  |  |  |  |  |  |
| *No* | 92.8 | 93.0 | 78.8 | 91.3 | 91.4 | 89.1 |
| *Yes* | 5.4 | 6.9 | 21.2 | 4.5 | 7.5 | 8.9 |
| *Missing* | 1.8 | 0.1 | 0.0 | 4.2 | 1.0 | 2.0 |
|  |  |  |  |  |  |  |
| Difficulty making or maintaining friendships in last 30 days, % |  |  |  |  |  |  |
| *No* | 97.8 | 93.2 | 92.1 | 94.3 | 97.2 | 93.8 |
| *Yes* | 0.5 | 6.6 | 7.8 | 1.5 | 1.8 | 4.2 |
| *Missing* | 1.8 | 0.2 | 0.0 | 4.2 | 1.1 | 2.0 |
|  |  |  |  |  |  |  |
| Difficulty dealing with strangers in last 30 days, % |  |  |  |  |  |  |
| *No* | 93.3 | 93.2 | 85.7 | 93.8 | 96.9 | 91.9 |
| *Yes* | 4.8 | 6.5 | 14.3 | 2.0 | 1.8 | 5.9 |
| *Missing* | 1.9 | 0.3 | 0.0 | 4.2 | 1.3 | 2.2 |
|  |  |  |  |  |  |  |
| Difficulty in sitting for long periods in last 30 days, % |  |  |  |  |  |  |
| *No* | 96.9 | 92.1 | 85.5 | 90.1 | 94.4 | 93.9 |
| *Yes* | 1.0 | 7.7 | 14.4 | 5.3 | 4.3 | 4.1 |
| *Missing* | 2.1 | 0.2 | 0.1 | 4.6 | 1.3 | 2.1 |
|  |  |  |  |  |  |  |
| Difficulty in walking 100m in last 30 days, % |  |  |  |  |  |  |
| *No* | 96.9 | 92.4 | 84.5 | 82.5 | 92.3 | 89.3 |
| *Yes* | 1.0 | 7.2 | 14.5 | 12.5 | 6.3 | 8.5 |
| *Missing* | 2.1 | 0.4 | 1.0 | 5.0 | 1.4 | 2.2 |
|  |  |  |  |  |  |  |
| Difficulty in standing from sitting down in last 30 days, % |  |  |  |  |  |  |
| *No* | 97.2 | 90.1 | 86.3 | 86.0 | 92.2 | 90.5 |
| *Yes* | 0.8 | 9.6 | 13.4 | 9.7 | 6.5 | 7.3 |
| *Missing* | 2.0 | 0.3 | 0.2 | 4.4 | 1.3 | 2.2 |
|  |  |  |  |  |  |  |
| Difficulty in standing for long periods in last 30 days, % |  |  |  |  |  |  |
| *No* | 95.6 | 81.1 | 75.6 | 81.3 | 86.3 | 84.0 |
| *Yes* | 2.2 | 18.4 | 23.8 | 14.1 | 11.5 | 13.6 |
| *Missing* | 2.1 | 0.5 | 0.7 | 4.6 | 2.2 | 2.4 |
|  |  |  |  |  |  |  |
| Difficulty climbing stairs without resting in last 30 days, % |  |  |  |  |  |  |
| *No* | 91.4 | 76.8 | 63.6 | 72.5 | 86.5 | 78.5 |
| *Yes* | 4.7 | 21.2 | 27.6 | 19.0 | 10.9 | 16.9 |
| *Missing* | 3.9 | 2.0 | 8.8 | 8.5 | 2.5 | 4.6 |
|  |  |  |  |  |  |  |
| Difficulty stooping, crouching or kneeling in last 30 days, % |  |  |  |  |  |  |
| *No* | 94.9 | 87.4 | 83.6 | 73.0 | 82.9 | 83.4 |
| *Yes* | 3.0 | 12.1 | 15.9 | 22.3 | 15.0 | 14.4 |
| *Missing* | 2.1 | 0.5 | 0.5 | 4.7 | 2.1 | 2.2 |
|  |  |  |  |  |  |  |
| Difficulty picking things up in last 30 days, % |  |  |  |  |  |  |
| *No* | 97.5 | 96.0 | 95.8 | 91.0 | 96.5 | 93.1 |
| *Yes* | 0.5 | 3.8 | 4.1 | 4.7 | 2.1 | 4.9 |
| *Missing* | 2.0 | 0.2 | 0.1 | 4.3 | 1.3 | 2.1 |
| Difficulty taking care of household responsibilities in last 30 days, % |  |  |  |  |  |  |
| *No* | 95.0 | 92.4 | 84.8 | 89.1 | 91.9 | 90.9 |
| *Yes* | 1.7 | 6.8 | 13.7 | 5.6 | 6.6 | 6.7 |
| *Missing* | 3.3 | 0.8 | 1.5 | 5.4 | 1.5 | 2.4 |
|  |  |  |  |  |  |  |
| Difficulty joining in community activities in last 30 days, % |  |  |  |  |  |  |
| *No* | 95.4 | 86.0 | 86.5 | 88.1 | 80.9 | 90.4 |
| *Yes* | 1.4 | 12.5 | 9.6 | 5.2 | 8.6 | 6.8 |
| *Missing* | 3.2 | 1.5 | 4.0 | 6.7 | 10.5 | 2.9 |
|  |  |  |  |  |  |  |
| Difficulty extending arms above shoulder level in last 30 days, % |  |  |  |  |  |  |
| *No* | 97.2 | 96.8 | 94.2 | 90.4 | 94.0 | 93.7 |
| *Yes* | 0.7 | 3.0 | 5.7 | 5.4 | 4.7 | 4.1 |
| *Missing* | 2.0 | 0.2 | 0.1 | 4.2 | 1.3 | 2.2 |
| Difficulty concentrating on doing something for 10 mins in last 30 days, % |  |  |  |  |  |  |
| *No* | 97.1 | 96.2 | 89.7 | 92.4 | 96.6 | 92.6 |
| *Yes* | 0.8 | 3.3 | 10.0 | 3.2 | 2.0 | 5.3 |
| *Missing* | 2.1 | 0.5 | 0.3 | 4.4 | 1.4 | 2.1 |
|  |  |  |  |  |  |  |
| Difficulty walking a long distance in last 30 days, % |  |  |  |  |  |  |
| *No* | 93.5 | 79.4 | 69.3 | 71.6 | 75.0 | 79.0 |
| *Yes* | 4.0 | 19.6 | 28.0 | 21.9 | 19.8 | 18.6 |
| *Missing* | 2.5 | 0.9 | 2.7 | 6.5 | 5.2 | 2.4 |
|  |  |  |  |  |  |  |
| Difficulty washing whole body in last 30 days, % |  |  |  |  |  |  |
| *No* | 97.1 | 97.4 | 96.7 | 91.8 | 94.3 | 96.2 |
| *Yes* | 0.8 | 2.2 | 3.2 | 3.9 | 3.9 | 1.8 |
| *Missing* | 2.1 | 0.4 | 0.2 | 4.3 | 1.8 | 2.0 |
|  |  |  |  |  |  |  |
| Difficulty getting dressed in last 30 days, % |  |  |  |  |  |  |
| *No* | 97.5 | 97.9 | 97.3 | 91.1 | 96.8 | 96.7 |
| *Yes* | 0.5 | 1.8 | 2.6 | 4.5 | 1.9 | 1.3 |
| *Missing* | 2.0 | 0.3 | 0.1 | 4.4 | 1.3 | 2.0 |
|  |  |  |  |  |  |  |
| Difficulty with day to day work in last 30 days, % |  |  |  |  |  |  |
| *No* | 94.1 | 90.0 | 90.5 | 87.8 | 93.0 | 93.5 |
| *Yes* | 1.3 | 9.0 | 9.0 | 5.3 | 5.2 | 4.4 |
| *Missing* | 4.6 | 1.0 | 0.5 | 6.9 | 1.8 | 2.1 |
|  |  |  |  |  |  |  |
| Difficulty carrying things in last 30 days, % |  |  |  |  |  |  |
| *No* | 89.7 | 74.3 | 77.8 | 62.5 | 87.7 | 88.5 |
| *Yes* | 6.6 | 23.8 | 20.8 | 27.5 | 9.4 | 8.8 |
| *Missing* | 3.7 | 1.9 | 1.4 | 10.0 | 2.9 | 2.7 |
|  |  |  |  |  |  |  |
| Difficulty moving around inside your home in last 30 days, % |  |  |  |  |  |  |
| *No* | 97.5 | 96.3 | 96.1 | 90.4 | 96.4 | 95.6 |
| *Yes* | 0.5 | 3.3 | 3.6 | 5.2 | 2.0 | 2.2 |
| *Missing* | 2.0 | 0.4 | 0.3 | 4.5 | 1.6 | 2.1 |
| Difficulty with eating in last 30 days, % |  |  |  |  |  |  |
| *No* | 97.5 | 98.7 | 95.1 | 93.1 | 97.0 | 96.8 |
| *Yes* | 0.5 | 1.1 | 3.8 | 2.6 | 1.0 | 1.0 |
| *Missing* | 2.0 | 0.2 | 1.1 | 4.3 | 1.9 | 2.2 |
|  |  |  |  |  |  |  |
| Difficulty with getting up from lying down in last 30 days, % |  |  |  |  |  |  |
| *No* | 97.4 | 95.8 | 95.1 | 91.2 | 92.2 | 93.0 |
| *Yes* | 0.6 | 3.9 | 4.8 | 4.5 | 6.2 | 4.9 |
| *Missing* | 2.0 | 0.3 | 0.1 | 4.3 | 1.6 | 2.1 |
|  |  |  |  |  |  |  |
| Difficulty getting to and using toilet in last 30 days, % |  |  |  |  |  |  |
| *No* | 97.3 | 96.5 | 95.0 | 92.9 | 96.3 | 95.4 |
| *Yes* | 0.6 | 3.2 | 4.8 | 2.8 | 1.8 | 2.2 |
| *Missing* | 2.1 | 0.3 | 0.3 | 4.3 | 1.9 | 2.4 |
| Difficulty in getting to place to place via public or private transport in last 30 days, % |  |  |  |  |  |  |
| *No* | 95.6 | 95.0 | 82.6 | 84.8 | 89.0 | 91.6 |
| *Yes* | 1.9 | 4.4 | 14.0 | 9.2 | 7.9 | 5.9 |
| *Missing* | 2.4 | 0.5 | 3.5 | 6.0 | 3.1 | 2.5 |
|  |  |  |  |  |  |  |
| Difficulty getting out of your home in last 30 days, % |  |  |  |  |  |  |
| *No* | 96.6 | 95.5 | 88.5 | 86.0 | 94.0 | 92.7 |
| *Yes* | 1.1 | 4.1 | 10.6 | 8.3 | 3.5 | 4.7 |
| *Missing* | 2.3 | 0.4 | 0.9 | 5.7 | 2.4 | 2.6 |
|  |  |  |  |  |  |  |
| Been emotionally affected by health in last 30 days, % |  |  |  |  |  |  |
| *No* | 96.5 | 93.2 | 87.6 | 89.1 | 83.7 | 85.1 |
| *Yes* | 1.5 | 6.4 | 12.2 | 6.4 | 14.7 | 7.7 |
| *Missing* | 2.1 | 0.4 | 0.2 | 4.5 | 1.6 | 7.2 |
|  |  |  |  |  |  |  |
| **Mental health** |  |  |  |  |  |  |
| Good mental health  *No*  *Yes*  *Missing* | 1.8  95.4  2.8 | 8.5  90.9  0.6 | 17.8  82.0  0.2 | 16.5  79.2  4.3 | 9.3  88.9  1.8 | 5.1  90.1  4.8 |
|  |  |  |  |  |  |  |
| Depression diagnosis, % |  |  |  |  |  |  |
| *No* | 97.8 | 98.0 | 95.5 | 84.5 | 94.0 | 92.4 |
| *Yes* | 0.3 | 1.6 | 4.4 | 11.3 | 4.5 | 2.9 |
| *Missing* | 1.9 | 0.4 | 0.0 | 4.2 | 1.5 | 4.7 |
|  |  |  |  |  |  |  |
| ICD-10 depression, % |  |  |  |  |  |  |
| *No* | 95.6 | 91.5 | 85.2 | 86.7 | 91.8 | 91.6 |
| *Yes* | 1.6 | 7.9 | 14.6 | 8.8 | 6.3 | 3.4 |
| *Missing* | 2.8 | 0.6 | 0.2 | 4.6 | 1.9 | 5.0 |
|  |  |  |  |  |  |  |

**Supplementary Table 3.:**Comparison of descriptive characteristics for complete case and full sample by country

[See: supplementary_table_3.xlsx]

**Supplementary Table 4:** Survey weighted log-binomial regression models of associations between fruit and vegetable intake and successful ageing (no/yes)

|  | **CHINA**  **(N = 8764)** | **GHANA**  **(N =2748)** | **INDIA**  **(N = 5182)** | **MEXICO**  **(N = 961)** | **RUSSIA**  **(N = 1244)** | **SOUTH AFRICA**  **(N = 1917)** |
| --- | --- | --- | --- | --- | --- | --- |
|  | **PR (95% CI), p** | **PR (95% CI), p** | **PR (95% CI), p** | **PR (95% CI), p** | **PR (95% CI), p** | **PR (95% CI), p** |
| **Unadjusted** |  |  |  |  |  |  |
| **Model 1a**  Servings of fruits consumed on typical day | 1.035 (1.007, 1.065)  0.015 | 0.837 (0.731, 0.958)  0.010 | 1.152 (0.985, 1.348)  0.077 | 1.279 (0.957, 1.708)  0.096 | 1.123 (0.885, 1.426)  0.336 | 0.918 (0.754, 1.117)  0.389 |
| **Model 2a**  Servings of vegetables consumed on typical day | 0.970 (0.951, 0.988)  0.002 | 0.965 (0.800, 1.164)  0.710 | 1.179 (1.017, 1.367)  0.029 | 1.450 (1.051, 2.001)  0.024 | 0.941 (0.740, 1.197)  0.617 | 0.893 (0.727, 1.096)  0.278 |
| **Model 3a**  Servings of fruits and vegetables consumed on a typical day | 0.987 (0.973, 1.001)  0.071 | 0.899 (0.822, 0.983)  0.019 | 1.120 (1.030, 1.219)  0.008 | 1.242 (1.036, 1.488)  0.019 | 1.025 (0.877, 1.197)  0.758 | 0.940 (0.834, 1.058)  0.302 |
| **Adjusted^*^** |  |  |  |  |  |  |
| **Model 1b**  Servings of fruits consumed on typical day | 1.002 (0.976, 1.029)  0.867 | 0.871 (0.775, 0.979)^a^  0.020 | 0.965 (0.824, 1.128)  0.651 | 1.154 (0.867, 1.535)  0.326 | 1.080 (0.870, 1.340)^a^  0.485 | 0.873 (0.712, 1.070)  0.190 |
| **Model 2b**  Servings of vegetables consumed on typical day | 0.965 (0.947, 0.984)^a^  <0.001 | 0.974 (0.819, 1.159)  0.770 | 0.977 (0.848, 1.126)  0.748 | 1.367 (0.955, 1.958)  0.088 | 0.921 (0.751, 1.129)^a^  0.425 | 0.859 (0.697, 1.058)  0.153 |
| **Model 3b**  Servings of fruit and vegetables consumed on typical day | 0.977 (0.964, 0.991)^a^  0.002 | 0.915 (0.842, 0.995)^a^  0.038 | 0.978 (0.898, 1.065)  0.609 | 1.172 (0.969, 1.418)  0.101 | 0.999 (0.882, 1.132)^a^  0.990 | 0.911 (0.804, 1.032)  0.143 |
| **Adjusted^**^** |  |  |  |  |  |  |
| **Model 1c**  Servings of fruits consumed on typical day | 1.018 (0.991, 1.045)^a^  0.040 | 0.884 (0.787, 0.993)^a^  0.038 | 0.918 (0.767, 1.099)  0.350 | 1.167 (0.902, 1.509)  0.238 | 0.977 (0.773, 1.236)^a^  0.847 | 0.845 (0.685, 1.043)^a^  0.117 |
| **Model 2c**  Servings of vegetables consumed on typical day | 0.969 (0.952, 0.987)^a^  0.001 | 0.994 (0.829, 1.191)^a^  0.944 | 0.955 (0.824, 1.106)  0.535 | 1.416 (0.977, 2.052)  0.066 | 0.867 (0.695, 1.081)^a^  0.203 | 0.829 (0.673, 1.020)^a^  0.076 |
| **Model 3c**  Servings of fruit and vegetables consumed on typical day | 0.983 (0.971, 0.996)^a^  0.011 | 0.925 (0.848, 1.009)^a^  0.079 | 0.951 (0.863, 1.048)  0.308 | 1.181 (0.998, 1.398)  0.052 | 0.941 (0.820, 1.079)^a^  0.380 | 0.893 (0.789, 1.011)^a^  0.074 |

PR=Prevalence Ratio; CI=Confidence Interval. *First adjusted model includes age, sex, marital status, education, employment status, urban/rural location and ethnicity (apart from Mexico where ethnicity was not reported by most participants) which were identified as the minimal sufficient adjustment set to account for confounders. **Second adjusted model also includes smoking status, alcohol intake, BMI, total physical activity and sedentary time. ^a^ Log-binomial regression failed to converge; Poisson regression used instead to estimate risk ratios.

**Supplementary Table 5:** Survey weighted Poisson regression models of associations between fruit and vegetable intake and successful ageing score

|  | **CHINA**  **(N = 8764)** | **GHANA**  **(N =2748)** | **INDIA**  **(N = 5182)** | **MEXICO**  **(N = 961)** | **RUSSIA**  **(N = 1244)** | **SOUTH AFRICA**  **(N = 1917)** |
| --- | --- | --- | --- | --- | --- | --- |
|  | **IRR (95% CI), p** | **IRR (95% CI), p** | **IRR (95% CI), p** | **IRR (95% CI), p** | **IRR (95% CI), p** | **IRR (95% CI), p** |
| **Unadjusted** |  |  |  |  |  |  |
| **Model 1a**  Servings of fruits consumed on typical day | 0.962 (0.947, 0.977)  < 0.001 | 1.009 (0.990, 1.028)  0.350 | 0.961 (0.924, 0.999)  0.046 | 0.961 (0.902, 1.024)  0.214 | 0.931 (0.851, 1.018)  0.118 | 1.025 (0.971, 1.081)  0.375 |
| **Model 2a**  Servings of vegetables consumed on typical day | 1.009 (1.000, 1.017)  0.047 | 1.016 (0.978, 1.054)  0.418 | 0.925 (0.893, 0.958)  < 0.001 | 0.926 (0.878, 0.976)  0.005 | 0.994 (0.916, 1.080)  0.892 | 1.069 (1.024, 1.115)  0.002 |
| **Model 3a**  Servings of fruits and vegetables consumed on a typical day | 0.998 (0.992, 1.004)  0.558 | 1.008 (0.993, 1.023)  0.282 | 0.956 (0.933, 0.979)  < 0.001 | 0.959 (0.925, 0.994)  0.023 | 0.972 (0.927, 1.018)  0.230 | 1.029 (1.002, 1.057)  0.035 |
| **Adjusted^*^** |  |  |  |  |  |  |
| **Model 1b**  Servings of fruits consumed on typical day | 0.985 (0.972, 0.998)  0.027 | 1.007 (0.989, 1.026)  0.437 | 1.000 (0.965, 1.035)  0.989 | 0.980 (0.919, 1.045)  0.538 | 0.957 (0.880, 1.040)  0.298 | 1.043 (0.997, 1.090)  0.065 |
| **Model 2b**  Servings of vegetables consumed on typical day | 1.013 (1.006, 1.020)  < 0.001 | 1.014 (0.980, 1.049)  0.410 | 0.973 (0.938, 1.009)  0.144 | 0.939 (0.894, 0.987)  0.013 | 1.049 (0.978, 1.125)  0.182 | 1.078 (1.040, 1.118)  < 0.001 |
| **Model 3b**  Servings of fruit and vegetables consumed on typical day | 1.007 (1.002, 1.011)  0.007 | 1.007 (0.993, 1.021)  0.338 | 0.989 (0.966, 1.013)  0.376 | 0.970 (0.937, 1.005)  0.088 | 1.003 (0.961, 1.047)  0.890 | 1.037 (1.015, 1.060)  0.001 |
| **Adjusted^**^** |  |  |  |  |  |  |
| **Model 1c**  Servings of fruits consumed on typical day | 0.979 (0.967, 0.992)  0.002 | 1.011 (0.992, 1.031)  0.240 | 1.010 (0.972, 1.049)  0.613 | 0.984 (0.926, 1.046)  0.612 | 0.976 (0.896, 1.063)  0.568 | 1.036 (0.990, 1.084)  0.130 |
| **Model 2c**  Servings of vegetables consumed on typical day | 1.013 (1.006, 1.020)  < 0.001 | 1.018 (0.984, 1.053)  0.299 | 0.977 (0.941, 1.015)  0.234 | 0.946 (0.906, 0.988)  0.012 | 1.062 (0.996, 1.132)  0.065 | 1.076 (1.035, 1.119)  < 0.001 |
| **Model 3c**  Servings of fruit and vegetables consumed on typical day | 1.006 (1.001, 1.010)  0.019 | 1.010 (0.995, 1.025)  0.183 | 0.994 (0.969, 1.021)  0.994 (0.969, 1.021)  0.672 | 0.974 (0.944, 1.006)  0.105 | 1.015 (0.971, 1.061)  0.516 | 1.035 (1.011, 1.059)  0.004 |

IRR=Incidence Rate Ratio; CI=Confidence Interval. *First adjusted model includes age, sex, marital status, education, employment status, urban/rural location and ethnicity (apart from Mexico where ethnicity was not reported by most participants) which were identified as the minimal sufficient adjustment set to account for confounders. **Second adjusted model also includes smoking status, alcohol intake, BMI, total physical activity and sedentary time.

**Supplementary Figure 1:** Directed Acyclic Graph Primary Adjustment Model


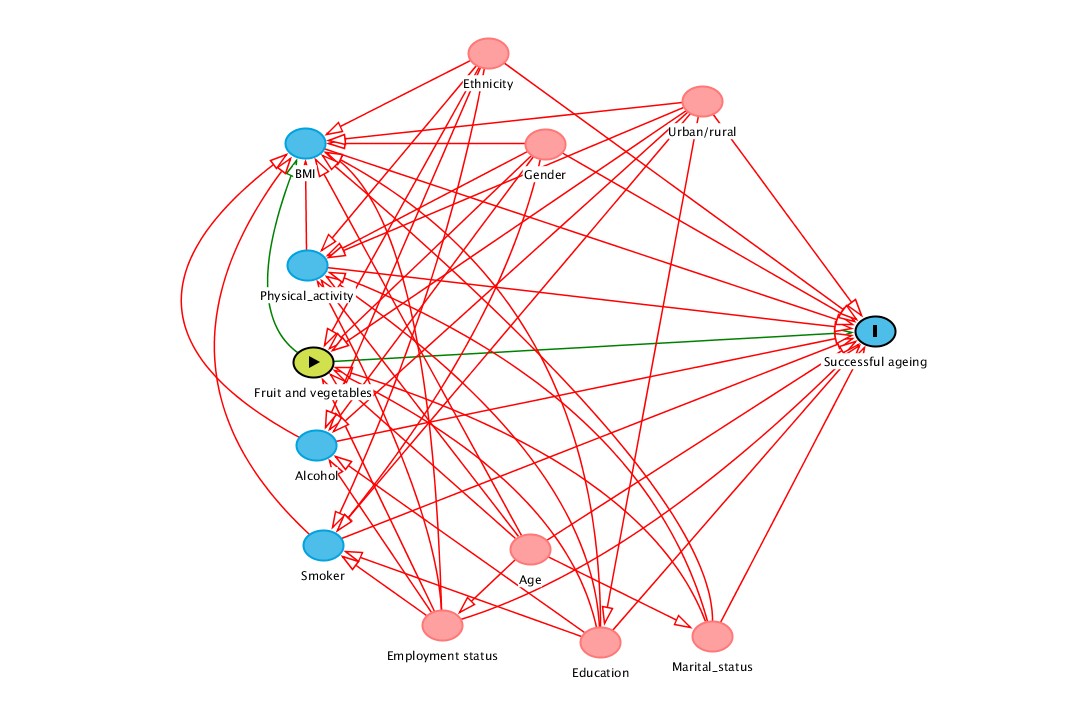


**Supplementary Figure 2:** Directed Acyclic Graph Secondary Adjustment Model with Lifestyle Variables Added


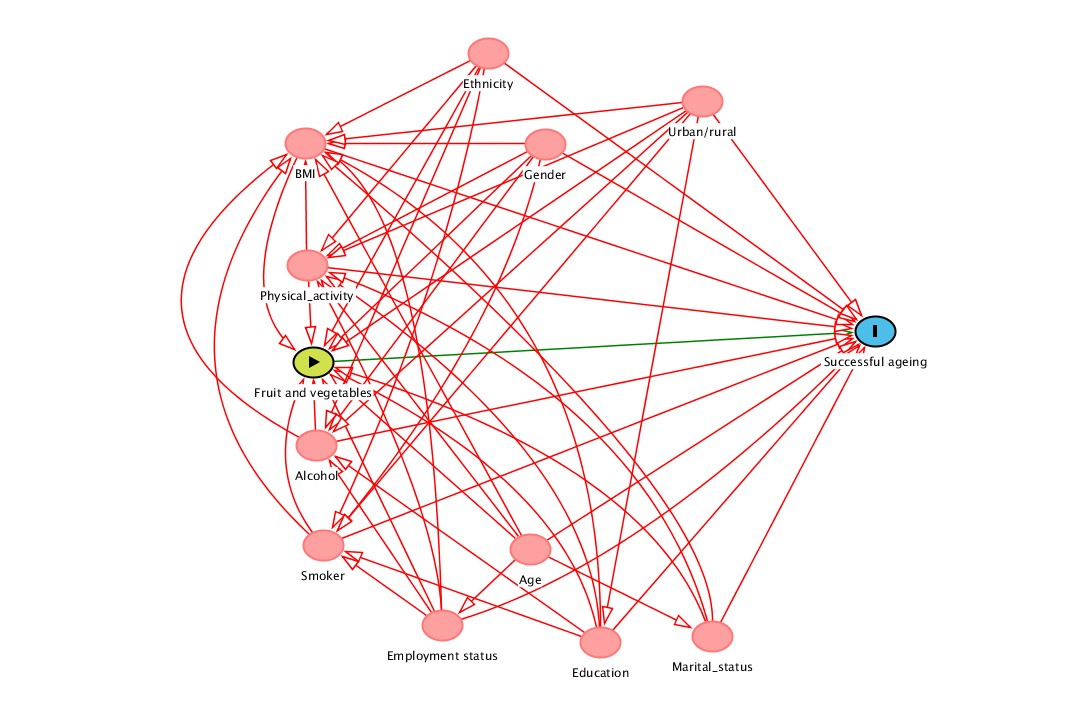


**Data availability**

Data is available from WHO on request upon completion of a Users Agreement available through WHO's SAGE website (www.who.int/healthinfo/systems/sage) and WHO's archive using the National Data Archive application (http://apps.who.int/healthinfo/systems/surveydata).

**REFERENCES**

1. Lamb, S.E. and D.J. Keene, *Measuring physical capacity and performance in older people.* Best Pract Res Clin Rheumatol, 2017. **31**(2): p. 243-254.

2. Oh-Park, M., et al., *Conventional and robust quantitative gait norms in community-dwelling older adults.* J Am Geriatr Soc, 2010. **58**(8): p. 1512-8.

3. Cruz-Jentoft, A.J., et al., *Sarcopenia: European consensus on definition and diagnosis: Report of the European Working Group on Sarcopenia in Older People.* Age Ageing, 2010. **39**(4): p. 412-23.

4. Tyrovolas, S., et al., *Factors associated with skeletal muscle mass, sarcopenia, and sarcopenic obesity in older adults: a multi-continent study.* J Cachexia Sarcopenia Muscle, 2016. **7**(3): p. 312-21.

5. World Health Organization, *A global brief on hypertension*. 2013, World Health Organisation: Geneva, Switzerland.

6. World Health Organization, *The ICD-10 classification of mental and behavioural disorders: Diagnostic criteria for research*. 1993: Geneva, Switzerland.

7. Kulkarni, R.S. and R.L. Shinde, *Depression and Its Associated Factors in Older Indians: A Study Based on Study of Global Aging and Adult Health (SAGE)-2007.* J Aging Health, 2015. **27**(4): p. 622-49.

8. Anand, A., *Understanding depression among older adults in six low-middle income countries using WHO-SAGE survey.* Behav Health, 2015. **1**((2)): p. 1-11.

9. Gopinath, B., et al., *Adherence to Dietary Guidelines and Successful Aging Over 10 Years.* J Gerontol A Biol Sci Med Sci, 2016. **71**(3): p. 349-55.

10. Bull, F.C., T.S. Maslin, and T. Armstrong, *Global physical activity questionnaire (GPAQ): nine country reliability and validity study.* J Phys Act Health, 2009. **6**(6): p. 790-804.
